# Supplementary material for: In the brain of the beholder: bi-stable motion reveals mesoscopic-scale feedback modulation in V1
Source: Brain Struct Funct. 2025 Apr 5;230(3):47. doi: 10.1007/s00429-025-02906-8 (PMC11972204; doi:10.1007/s00429-025-02906-8)
Supplement: Supplementary file 1 — (pdf 3425 KB) [file 429_2025_2906_MOESM1_ESM.pdf]

8 Supplementary Material

	Horizontal	Vertical
Duration (s)	6.29 ± 1.62	6.29 ± 1.34
Total Number of trials	1223	1204

Table 1: Report on perceptual horizontal and vertical trials during the ambiguous motion condition. Trial duration is reported for both the horizontal and the vertical motion type at the group level (mean ± standard deviation). The total number of trials is computed by considering all the subjects and the fMRI runs.

	V1 Hor.	V1 Ver.	hMT+ Hor.	hMT+ Ver.
Ambiguous	20.41 ± 2.4	20.36± 1.57	21.42 ± 0.78	21.81 ± 1.21
Physical	20.45 ± 2.03	19.9± 1.37	21.97 ± 1.52	22.44 ± 2.07

Table 2: tSNR is reported for each region of interest at the group level (mean± standard deviation), for both the ambiguous and the physical motion condition. We computed the tSNR map for each participant and each pre-processed functional run (both the ambiguous and the physical motion condition) as a ratio between the mean of the time course divided by the standard deviation of the time course. Then, we computed the average tSNR across voxels belonging to the same region of interest for each participant and condition. Finally, we computed the mean tSNR (and the standard deviation) across participants.

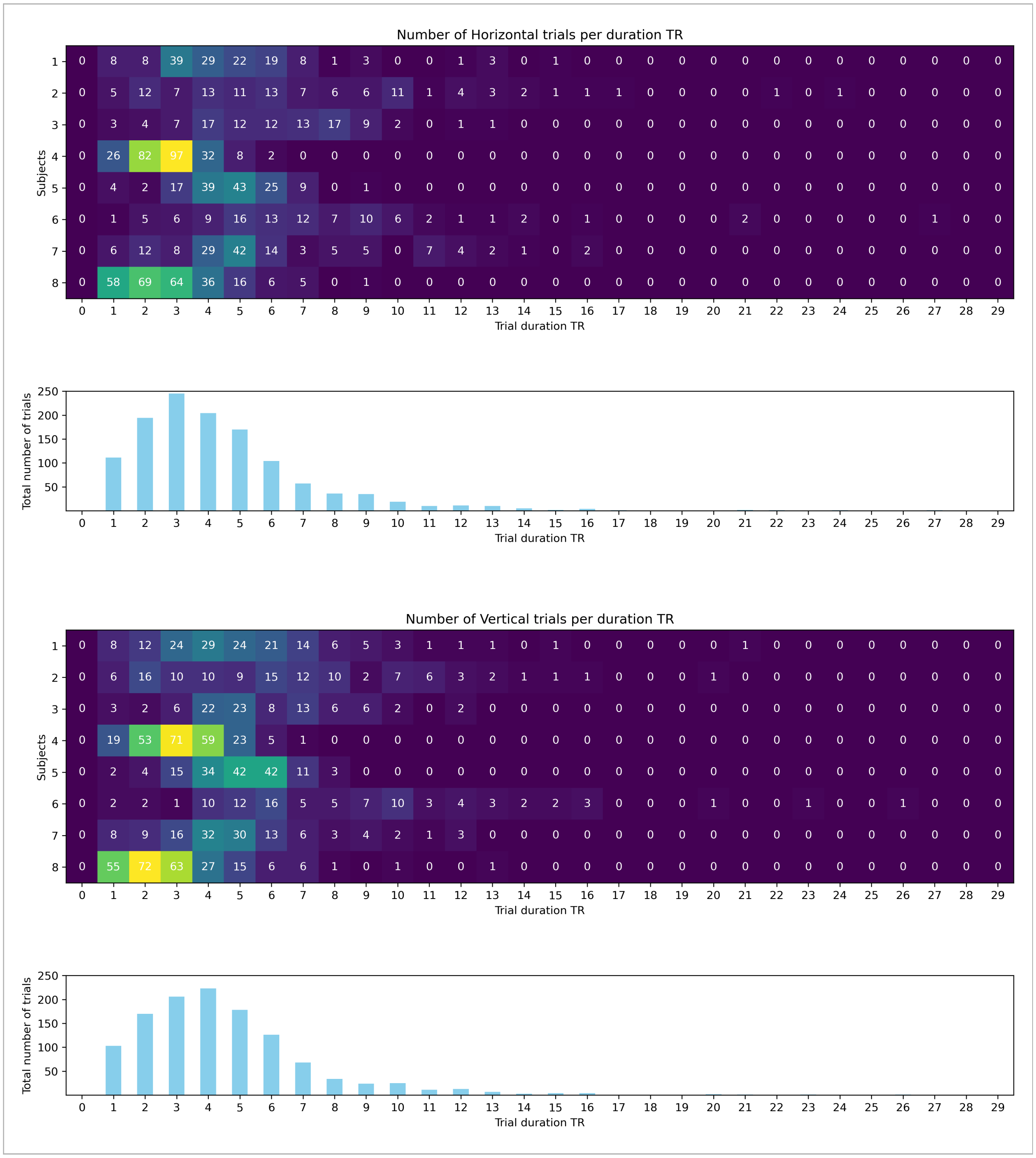

Supplementary Figure 1: Distribution of trial durations (horizontal and vertical) during the ambiguous motion condition for all the subjects.

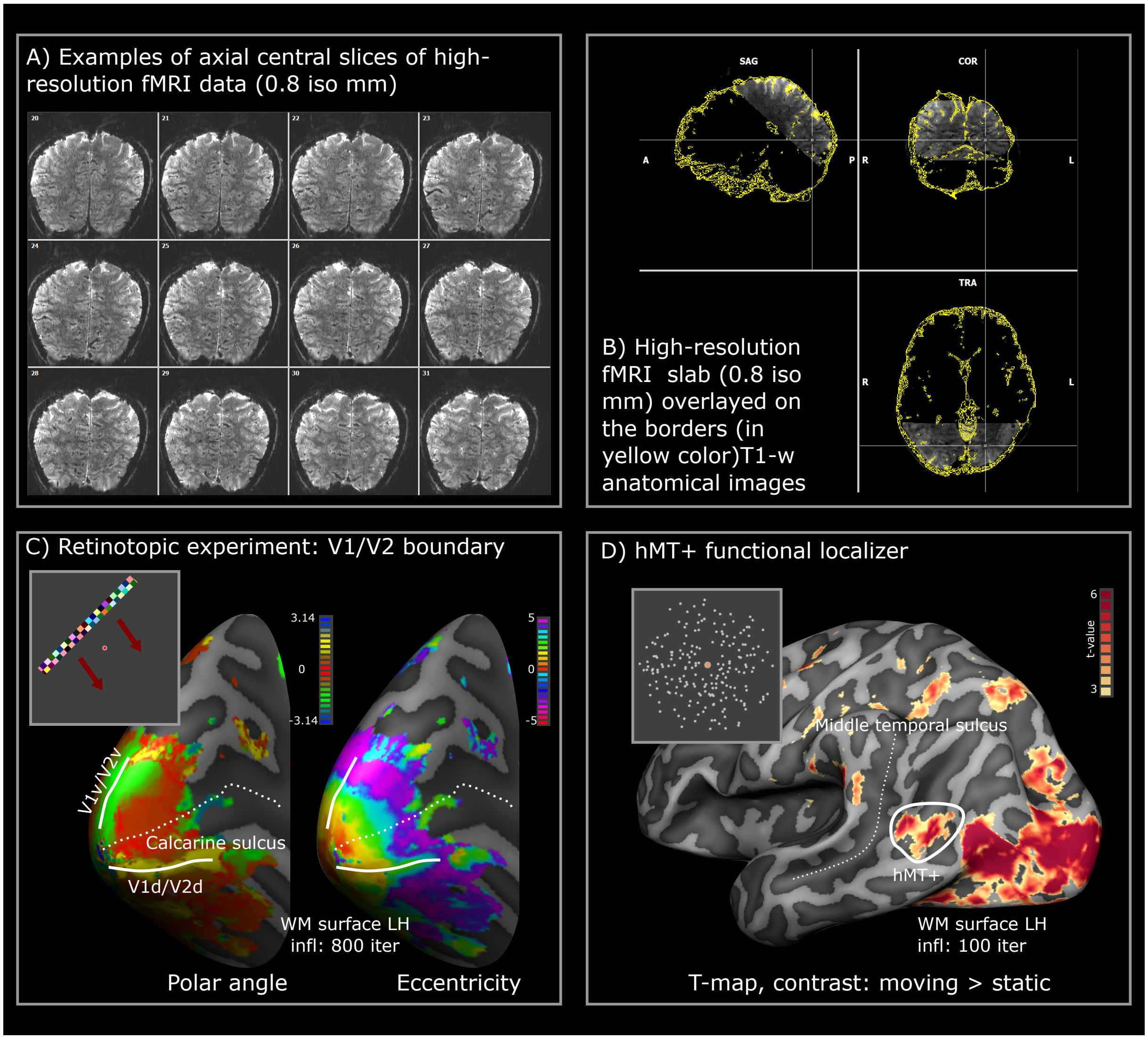

Supplementary Figure 2: Data quality and region of interest definition (sub-04). A) Examples of fMRI slices at 0.8 mm iso. B) High-resolution fMRI coverage. C) Example of polar angle and eccentricity maps obtained during the retinotopic experiment and manually drawn V1/V2 borders (white solid lines reported on the surface). D) Statistical t-map from hMT+ functional localizer run. White circle on the surface indicate hMT+ region of interest.

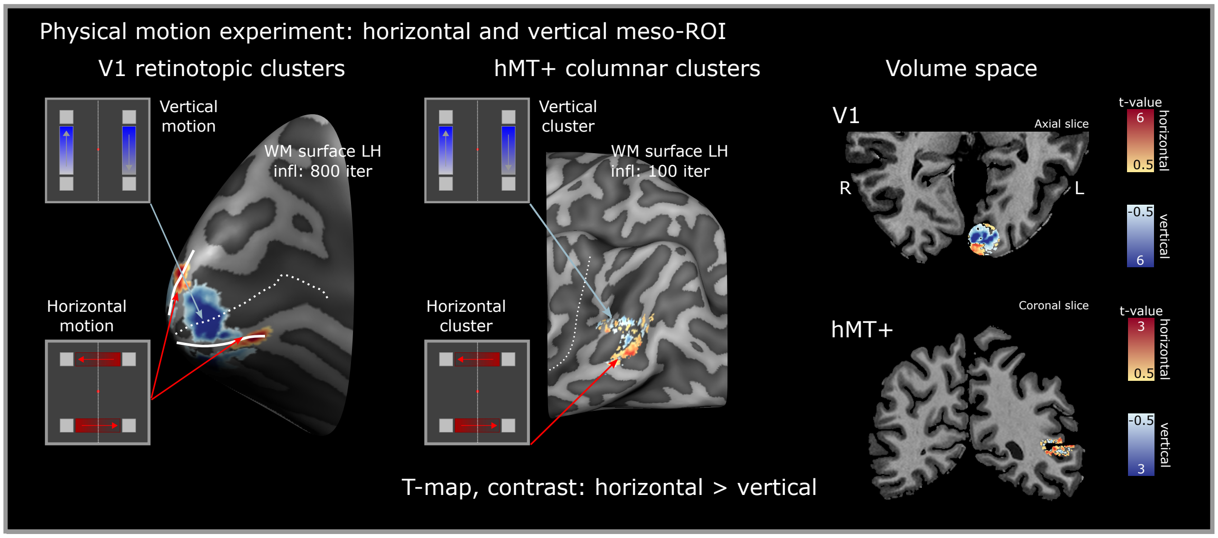

Supplementary Figure 3: Mesoscopic cluster definition (sub-04). Statistical t-maps (contrast: horizontal > vertical) from physical motion runs are used to define horizontal and vertical clusters (retinotopic clusters in V1, motion-specific clusters for hMT+). Maps are shown both in the surface and volume space on one exemplary slice.

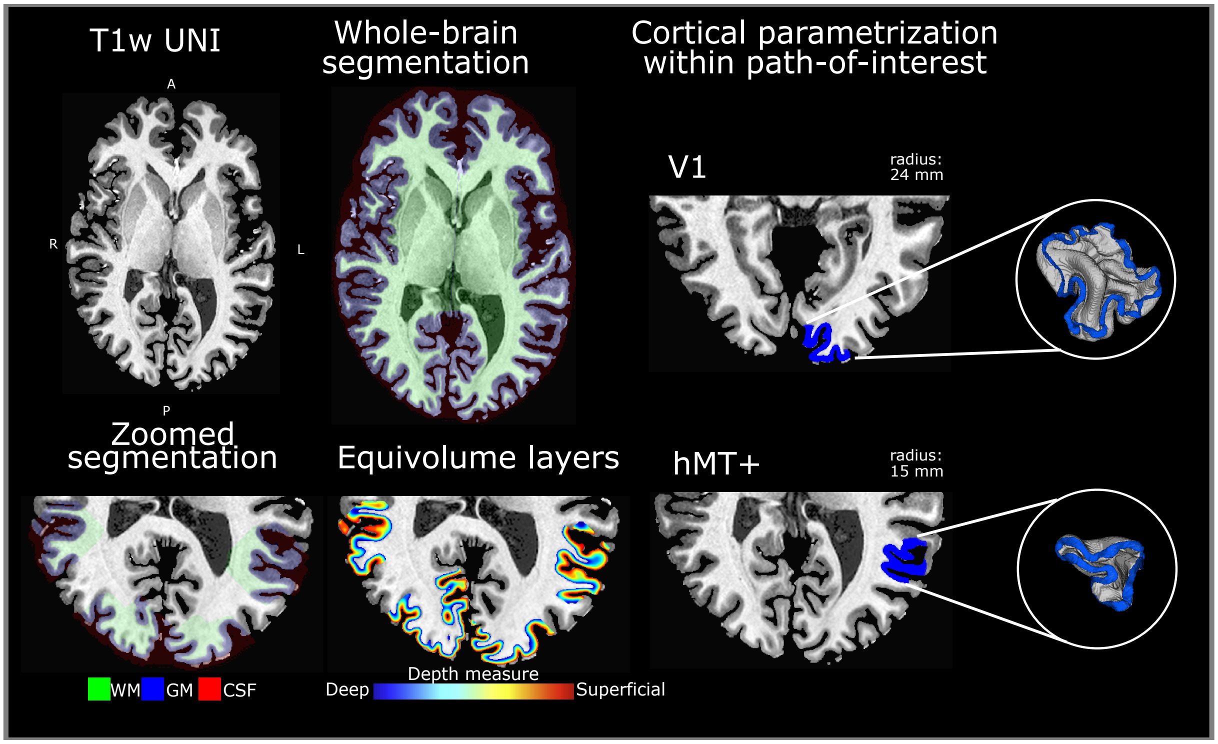

Supplementary Figure 4: Segmentation steps (sub-04). T1w UNI image from MP2RAGE is inputted in BrainVoyager advanced segmentation pipeline to obtain an initial whole brain segmentation. After manual editing, white matter segmentation was used to reconstruct the white matter surface used for drawing the regions of interest (V1/V2 borders, hMT+). Once delineated the ROIs, we further polished tissue segmentation around them ('Zoomed segmentation' step). Normalized equivolume depth-coordinate D and cortical layers are computed using `LN2_LAYERS` -equivol in LayNii. Finally, the `LN2_MULTILATERATE` program in LayNii is used to generate U,V cortical coordinates that together with D coordinate provide a whole 3D parametrization ('Cortical parametrization within patch of interest' step).
